# Allelochemical root-growth inhibitors in low-molecular-weight cress-seed exudate

**DOI:** 10.1093/aob/mcad200

**Published:** 2023-12-23

**Authors:** Muhammad Ishfaq Khan, Rifat Ara Begum, Lenka Franková, Stephen C Fry

**Affiliations:** The Edinburgh Cell Wall Group, Institute of Molecular Plant Sciences, The University of Edinburgh, Edinburgh EH9 3BF, UK; The Edinburgh Cell Wall Group, Institute of Molecular Plant Sciences, The University of Edinburgh, Edinburgh EH9 3BF, UK; The Edinburgh Cell Wall Group, Institute of Molecular Plant Sciences, The University of Edinburgh, Edinburgh EH9 3BF, UK; The Edinburgh Cell Wall Group, Institute of Molecular Plant Sciences, The University of Edinburgh, Edinburgh EH9 3BF, UK

**Keywords:** Allelochemicals, amaranth (*Amaranthus caudatus*), bioassay, chromatography, cress (*Lepidium sativum*), electrophoresis, hypocotyl elongation, potassium salts, root growth, seed exudate

## Abstract

**Background and Aims:**

Cress seeds release allelochemicals that over-stimulate the elongation of hypocotyls of neighbouring (potentially competing) seedlings and inhibit their root growth. The hypocotyl promoter is potassium, but the root inhibitor was unidentified; its nature is investigated here.

**Methods:**

Low-molecular-weight cress-seed exudate (LCSE) from imbibed *Lepidium sativum* seeds was fractionated by phase partitioning, paper chromatography, high-voltage electrophoresis and gel-permeation chromatography (on Bio-Gel P-2). Fractions, compared with pure potassium salts, were bioassayed for effects on *Amaranthus caudatus* seedling growth in the dark for 4 days.

**Key Results:**

The LCSE robustly promoted amaranth hypocotyl elongation and inhibited root growth. The hypocotyl inhibitor was non-volatile, hot acid stable, hydrophilic and resistant to incineration, as expected for K^+^. The root inhibitor(s) had similar properties but were organic (activity lost on incineration). The root inhibitor(s) remained in the aqueous phase (at pH 2.0, 6.5 and 9.0) when partitioned against butan-1-ol or toluene, and were thus hydrophilic. Activity was diminished after electrophoresis, but the remaining root inhibitors were neutral. They became undetectable after paper chromatography; therefore, they probably comprised multiple compounds, which separated from each other, in part, during fractionation. On gel-permeation chromatography, the root inhibitor co-eluted with hexoses.

**Conclusions:**

Cress-seed allelochemicals inhibiting root growth are different from the agent (K^+^) that over-stimulates hypocotyl elongation and the former probably comprise a mixture of small, non-volatile, hydrophilic, organic substances. Abundant components identified chromatographically and by electrophoresis in cress-seed exudate fitting this description include glucose, fructose, sucrose and galacturonic acid. However, none of these sugars co-chromatographed and co-electrophoresed with the root-inhibitory principle of LCSE, and none of them (in pure form at naturally occurring concentrations) inhibited root growth. We conclude that the root-inhibiting allelochemicals of cress-seed exudate remain unidentified.

## INTRODUCTION

Many plants are capable of adversely affecting the growth and development of neighbouring potential competitors, which is a phenomenon known as allelopathy (Molisch, 1937; Rice, 1984; Willis, 2007; He *et al.*, 2019) and attributed to ‘allelochemicals’. Some allelochemicals inhibit germination; others permit germination but interfere with the growth and development of ‘target’ seedlings. Such interference can include inhibition of growth or stimulation of excessive growth, which are adverse effects that could lead to the discovery of new herbicides. For example, Syngenta’s herbicide ‘Callisto’ contains mesotrione, an artificial analogue of the natural allelochemical leptospermone (Cornes, 2005; Dayan *et al.*, 2011; Cordeau *et al.*, 2016).

Allelopathic plants create and release allelochemicals that hinder the growth of surrounding roots (Asaduzzaman *et al.*, 2016; Yang and Kong, 2017), sometimes as an active response to chemicals from the presence of surrounding roots (Kong *et al.*, 2018; Li *et al.*, 2020). Conversely, the ‘target’ plant might detect the presence of the allelopathic species and modify root placement, thus avoiding the allelochemicals. The use of allelopathy has the potential to be a special tool for weed control and sustainable agriculture because it is natural and environmentally benign. Allelochemicals might be more biodegradable than conventional herbicides but might also have unfavourable impacts on species other than those targeted. This makes ecological studies necessary before widespread use (Khan and Khan, 2015).

Understanding allelochemicals is also important for an appreciation of the ecological factors influencing the success of different plants in diverse natural environments. Allelochemicals arise from various parts of the ‘donor’ plant, including root exudates (Rovira, 1969; Hale *et al.*, 1978; Curl and Truelove, 1986; Fan *et al.*, 1997; Uren, 2000) and root volatiles (Jassbi *et al.*, 2010), fallen leaves (Sarkar *et al.*, 2012; Sahu and Devkota, 2013; Szwed *et al.*, 2020), seeds (Hasegawa *et al.*, 1992; Higashinakasu *et al.*, 2004; Boydston *et al.*, 2011) and stems (Li *et al.*, 2016). As a classic example, black walnut (*Juglans nigra*), one of the best-known allelopathic plants, generates the extremely effective allelochemical juglone from fallen leaves (Lee and Campbell, 1969).

In this paper, we explore bioactive substances exuded from cress seeds (*Lepidium sativum*), which are known to interfere with the growth and development of neighbouring, potentially competing seedlings, such as those of amaranth (*Amaranthus caudatus*) and lettuce (*Lactuca sativa*) (Hasegawa *et al.*, 1992; Iqbal and Fry, 2012). Amaranth was chosen as the target species in the present work because of the small size of its seedlings, such that numerous replicates can be performed easily in small Petri dishes, and because amaranth has previously been shown in the above-cited literature to be sensitive to allelochemicals. Previous work has confirmed that many species, including *Arabidopsis thaliana*, *Helianthus annuus*, *Celosia cristata* and *Avena sativa*, are targeted by cress-seed exudate (Higashinakasu *et al.*, 2004).

Cress-seed exudate over-stimulates the elongation of amaranth hypocotyls and restricts their growth in girth while simultaneously inhibiting root growth (Hasegawa *et al.*, 1992; Yamada *et al.*, 2007; Iqbal *et al.*, 2012), and these effects would be detrimental to amaranth seedlings as model ‘competitors’. Originally, the main allelochemical present in cress seed(ling) exudate was suggested to be lepidimoic acid [4-deoxy-β-l-*threo*-hex-4-enopyranuronosyl-(1→2)-l-rhamnose, an unsaturated acidic disaccharide derived from the pectic domain rhamnogalacturonan-I] (Hasegawa *et al.*, 1992; Yamada *et al.*, 2007). Subsequent work challenged this idea (Iqbal *et al.*, 2016). One of the cress-seed allelochemicals (the one responsible for over-stimulation of hypocotyl elongation) was shown to be inorganic potassium, K^+^ (Fry, 2017), which promotes hypocotyl elongation in etiolated seedlings (McIntyre and Boyer, 1984). However, the active principle responsible for root growth inhibition was shown to be neither lepidimoic acid nor K^+^, but instead an as-yet unidentified organic material (separable from lepidimoic acid and destroyed by ashing; Fry, 2017). Our hypothesis was that the root-growth-inhibiting principle was one or more organic substances (most likely to be a mixture of components), which we have characterized, in part, in the present work.

## MATERIALS AND METHODS

### Materials

Volatile chromatography solvents and electrophoresis buffers, filter paper discs (47 mm; Whatman No. 1), chromatography paper (Whatman No. 3) and general laboratory chemicals were sourced as described previously (Fry, 2017). Plastic-backed silica-gel thin-layer chromatography (TLC) plates were from Merck (Darmstadt, Germany; https://www.merckgroup.com/en). Bio-Gel P-2 was from Bio-Rad (Hercules, CA, USA; https://www.bio-rad.com/en-uk/contact-us). Seeds, from E. W. King & Co. (Kelvedon, Colchester, Essex, UK; https://www.kingsseeds.com), were *Amaranthus caudatus* (code AMA001) and *Lepidium sativum* (Cress Fine Curled; code CRE03).

### Preparation of low-molecular-weight cress-seed exudate

Low-molecular-weight cress-seed exudate (LCSE) was prepared as described by Iqbal *et al.* (2016). Cress seeds (5 g dry weight) were placed in a dialysis sac with a total of 100 mL water (~50 mL inside the sac and 50 mL outside) for 48 h at 4 °C in the dark. The external solution (LCSE; ~50 mL; total dissolved solids ~1.6 mg mL^−1^) was filtered through filter paper and stored frozen. This was the concentration of LCSE used in all bioassay experiments unless stated otherwise.

The osmolality of LCSE was estimated at ~11 mOsmol kg^−1^ by use of a freezing-point depression osmometer (Micro-Digital Osmometer MOD200 Plus, Camlab, Cambridge, UK; Rosko *et al.*, 2017; see Supplementary Data Table S1).

### Properties of the bioactive principle(s) of LCSE

Fifteen independent preparations of LCSE were subjected to various treatments (1 mL LCSE for each treatment): freezing/thawing; freeze-drying; incubating in the presence of 0.25 m formic acid at 20 or 120 °C for 1 h; ashing in a glass tube held over a Bunsen flame for 3 min at ~400 °C; and partitioning between 20 mm aqueous formic acid (pH 2.7) and ethyl acetate. Controls included identical formic acid/water/ethyl acetate mixtures with no LCSE. All samples (except those that were simply frozen/thawed) were dried in a SpeedVac, re-dried several times from 0.1 mL water and, finally, redissolved in 1 mL water. Each solution was then applied to amaranth seeds (see ‘bioassay’).

### Bioassay

All 1-mL samples of treated LCSE (or fractions derived therefrom by chromatography or electrophoresis) were applied to two 4.7 cm discs of Whatman No. 1 filter paper in a 5 cm plastic Petri dish, and ten amaranth seeds (well spaced) were added. The lids were sealed with Parafilm and the dishes incubated in the dark at 25 °C for 4 days. The seedlings were stained with Aniline Blue (Long *et al.*, 2008; Fry, 2017), and the hypocotyls (white) and roots (stained blue) were measured with a ruler. The above bioassay procedure was also used in experiments investigating the biological effects of potassium salts and chromatography and electrophoresis solvents.

**Fig. 1.**
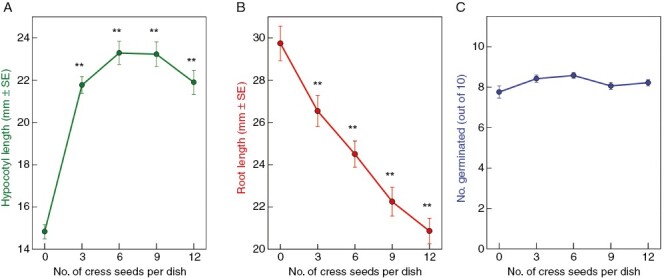
Effect of cress seed(ling)s on germination and seedling growth of amaranth. Amaranth seeds (ten per dish) were incubated in the dark on damp filter paper in 5-cm Petri dishes in the presence of 0–12 cress seeds. (A, B) After 4 days, the amaranth seedlings were measured (A, hypocotyls; B, roots). (C) The number of amaranth seeds that germinated is also recorded. *n* = 61–67 dishes for each number of cress seeds tested; asterisks indicate a significant effect of cress seeds, **P < 0.002.

**Fig. 2.**
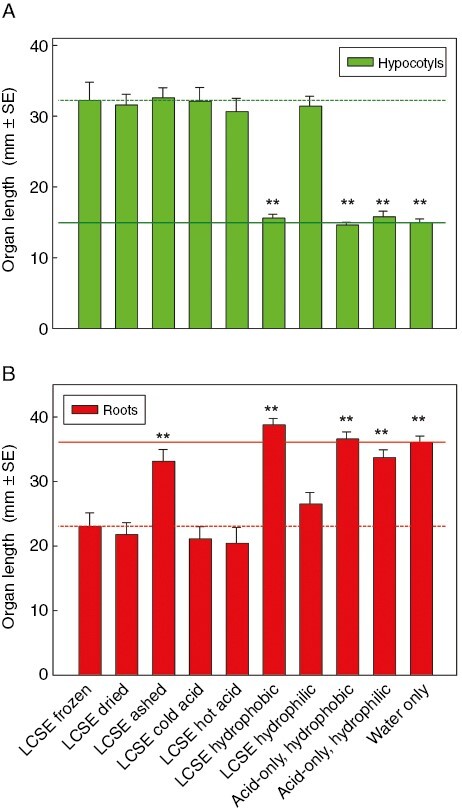
Low-molecular-weight cress-seed exudate (LCSE) promotes amaranth hypocotyl growth and inhibits amaranth root growth through two different agents. Amaranth seeds were incubated on damp filter paper for 4 days in the presence of LCSE (total solute concentration ~1.6 mg mL^−1^) that had been stored frozen, compared with LCSE treated by: freeze-drying; ashing in a Bunsen flame; incubation with 0.25 m formic acid for 1 h at 20 or 120 °C; or partitioning against ethyl acetate (acidified to pH 2.7 with 20 mm formic acid; the upper hydrophobic organic phase and lower hydrophilic aqueous phase were bioassayed separately). Any formic acid or ethyl acetate was dried off before the bioassays, and all LCSE specimens were reconstituted in deionized water to the original volume. The right-hand three bars represent controls with no LCSE present: ‘acid-only hydrophobic’ and ‘acid-only hydrophilic’ were the organic and aqueous phase, respectively, after 20 mm formic acid was shaken with ethyl acetate. *n* = 15 Petri dishes for each treatment; **P < 0.002 compared with frozen/thawed LCSE. Dashed line, frozen/thawed LCSE; solid line, water-only control.

**Fig. 3.**
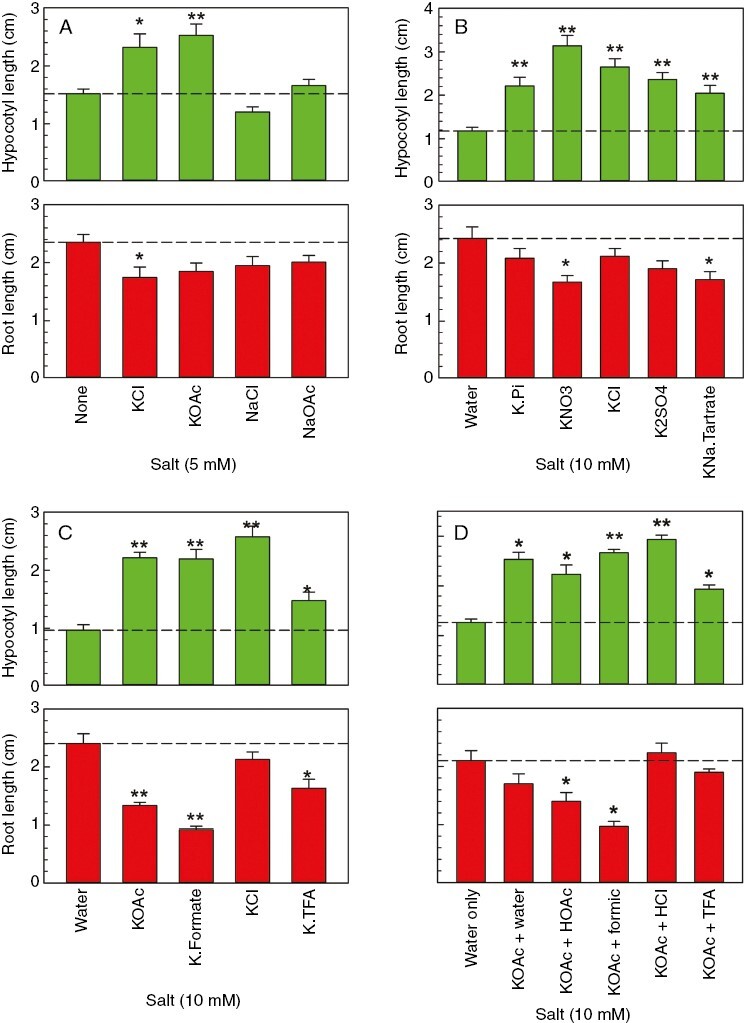
Effect of salts on amaranth seedling growth. Ten amaranth seeds per dish were incubated for 4 days in the dark in the presence of various salts at 5 mm (A) or 10 mm (B–D), then the hypocotyl and root lengths were measured. Each treatment was applied in three Petri dishes; the histograms show the mean ± s.e.m. organ lengths. In each case, the germination was 70–80 %. In C, the salts were prepared in house by adjustment of 10 mm KOH to pH 6.0 with the appropriate acid. In D, the 10 mm potassium acetate was adjusted to pH 3.0 with a small excess of acetic acid, mimicking a potential ‘K^+^-trapped’ anion present in low-molecular-weight cress-seed exudate, then dried from a 100 mm solution of the acid (or water) named on the *x*-axis. The dashed line indicates the water-only control. *P < 0.01, **P < 0.001 (in each histogram compared with the water-only control).

**Fig. 4.**
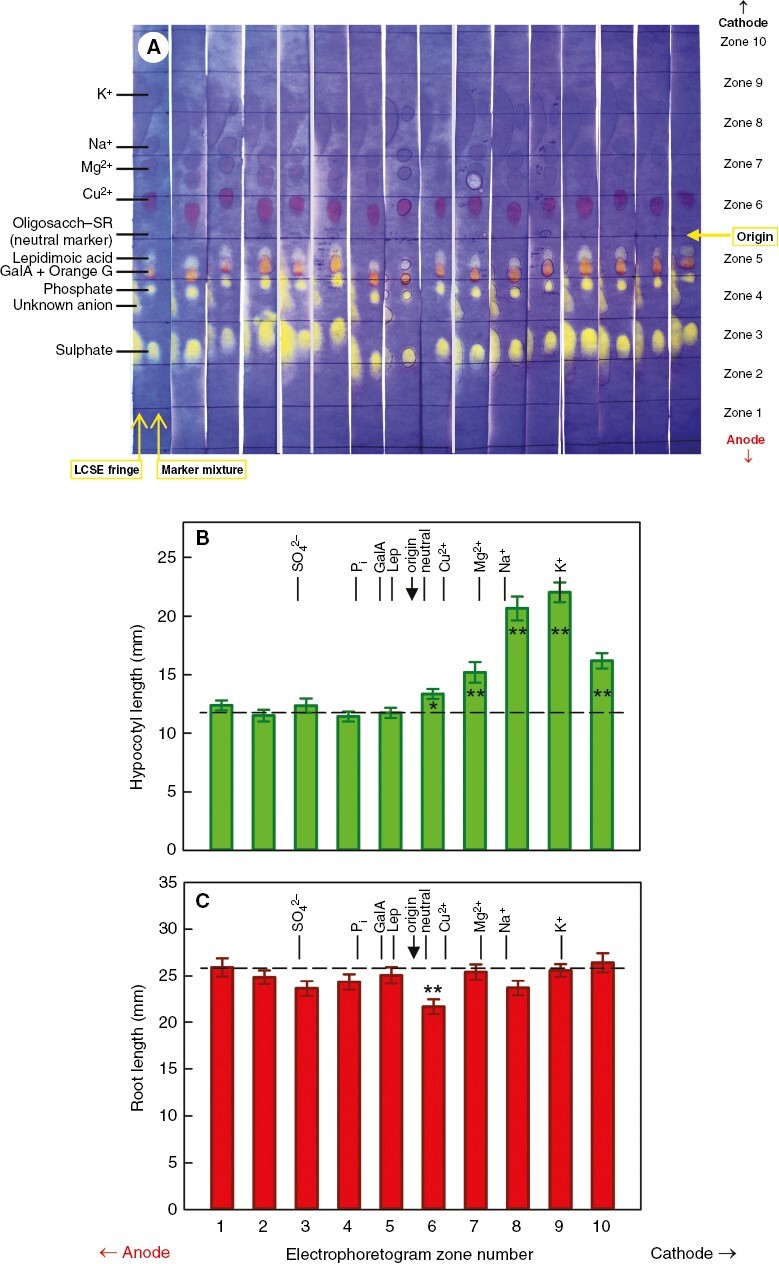
Behaviour of the active principles of low-molecular-weight cress-seed exudate (LCSE) on electrophoresis. Replicate samples (*n* = 48) of LCSE were fractionated by paper electrophoresis at pH 6.5 for 13 min at 2.5 kV. Each of the 48 electrophoretograms carried a marker mixture containing K^+^, Na^+^, Mg^2+^, Cu^2+^, XXXGol-sulphorhodamine (oligosacch–SR; a neutral marker, fluorescent), lepidimoic acid (Lep), galacturonic acid (GalA), Orange G, phosphate (P_i_) and sulphate (SO_4_^2−^). (A) The marker mixture was cut off each of the 48 electrophoretograms, together with a fringe of the neighbouring LCSE loading, and stained with Bromophenol Blue; 16 representative runs are illustrated. The unstained majority of each electrophoretogram was then cut into ten zones, each of which was eluted with water and the eluate bioassayed on hypocotyl growth (B) and root growth (C) of amaranth seedlings. The histograms show the mean seedling lengths (±s.e.m.; *n* ≈ 48). In B, the dashed line indicates the mean of the shortest four; in C, it is the mean of the longest four. Asterisks indicate that the specific zone differed significantly from the mean (*n* ≈ 192) of the relevant four zones: *P < 0.01, **P < 0.001.

**Fig. 5.**
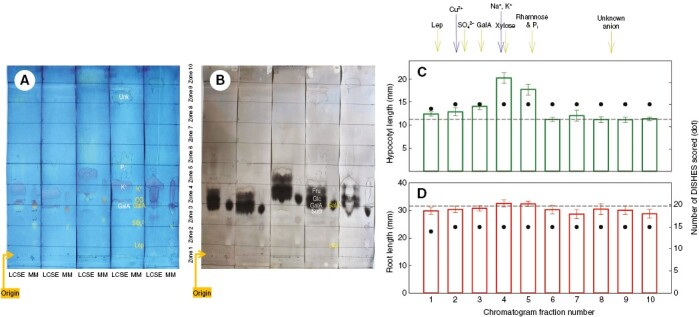
Paper chromatography of active principle(s) present in low-molecular-weight cress-seed exudate (LCSE). (A, B) Paper chromatography (in butan-1-ol/acetic acid/water) of five examples out of 15 independent LCSE samples: (A) stained with Bromophenol Blue, revealing ionic constituents; and (B) same chromatograms stained with silver nitrate, revealing sugars. Abbreviation: ‘LCSE’, a 100-µL streak loading (~4 cm × 1 cm) of 20-fold concentrated LCSE; MM, marker mixture [10 µL, containing 30 mm of each of lepidimoic acid, galacturonic acid, potassium sulphate and a trace of Orange G]. Spots labelled in yellow represent components of marker mixture. Spots labelled in white represent components of LCSE (Fru, fructose; GalA, galacturonic acid; Glc, glucose; Sucr, sucrose; Unk, unidentified anion). [Spots are labelled only on the fourth chromatogram.] Chromatography was on acid-washed Whatman No. 3 paper, developed in butan-1-ol/acetic acid/water (12:3:5) for 20 h. After thorough drying, the paper was dipped rapidly through methanol/acetone (1:2) and re-dried, repeated several times, and finally re-dried in a draught of air overnight, helping to remove the last traces of acetic acid. (C, D) Strips corresponding to zones 1–10 were excised from replicate 100-µL streak-loaded chromatograms (identical to A and B but not stained; not shown) of the 15 concentrated LCSE samples; each strip was eluted into 1 mL water, and the eluates were bioassayed for effects on the growth of amaranth hypocotyls (C) and roots (D). The approximate migration positions of various markers (with some variation between the 15 chromatograms) are indicated above histogram C.

**Fig. 6.**
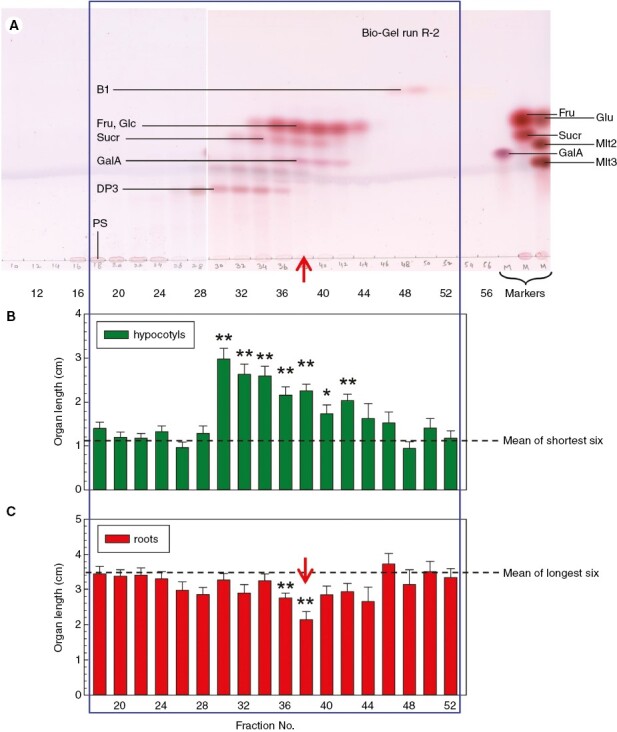
Behaviour of the active principles of low-molecular-weight cress-seed exudate (LCSE) on gel-permeation chromatography. The LCSE was run through a Bio-Gel P-2 column, and selected even-numbered fractions were tested for thymol-reactive sugars (A) and for the ability to promote amaranth hypocotyl growth (B) and to inhibit root growth (C). Abbreviations in A are as follows: B1, unidentified sugar as named by Iqbal *et al.* (2016); DP3, probable neutral trisaccharide; Fru, fructose; Glc, glucose; Mlt2, maltose; Mlt3, maltotriose; PS, polysaccharides; Sucr, sucrose. Asterisks indicate a significant effect of the fraction (mean ± s.e.m.; *n* ≈ 20) compared with the mean of all fractions; *P < 0.01, **P < 0.001. The most effective fraction for root inhibition is marked with a vertical arrow.

Each bioassay was conducted in at least three replicate Petri dishes. Experiments were also replicated: the data in Fig. 1 came from three complete repeats of the experiment conducted in 2019, 2022 and 2023, with the work in each year comprising 20–23 independently set up dishes; the data in Fig. 2 were also from three separate years, each with 15–22 independent LCSE preparations; data in Fig. 3 comprised four independent experiments (shown) plus an equal number of repeat runs; data in Figs 4 and 5 were from three and two years’ work, respectively, each performed with 15–20 independent LCSE preparations; and Fig. 6 shows one representative experiment, with two additional repeats being shown in the Supplementary data.

### Chromatography and electrophoresis

Whatman No. 3 papers (46 cm × 57 cm sheets) were washed by irrigation (as if for paper chromatography by the descending method) in 5 % formic acid for 2 days, then in water for a further 5 days. The washing removed traces of ionic substances present in the paper. For paper chromatography, 100 µL of a 20-fold concentrated preparation of LCSE was applied as a 4 cm × 1 cm streak to 57-cm-long sheets of acid-washed Whatman No. 3 paper and developed by the descending method in butan-1-ol/acetic acid/water (BAW; 12:3:5) for 20 h. The paper was dried and re-dried, and fractions were eluted, as above.

For high-voltage paper electrophoresis, similar LCSE samples were applied to 57-cm-long sheets of acid-washed Whatman No. 3 paper (with the LCSE samples loaded about halfway between the anode and cathode), which was then wetted with a volatile buffer [pyridine/acetic acid/H_2_O (33:1:300 v/v/v), pH 6.5] at 2.5 kV for 13 min. The buffer was then removed in a stream of air, and the paper was re-dried, then repeatedly dipped through acetone/methanol (2:1) and re-dried; strips of the paper were then eluted with water, and the eluate was freeze-dried and re-dissolved in water.

Ionic marker compounds, and LCSE tracks that were not going to be bioassayed, were stained by rapid dipping through Bromophenol Blue (0.4 g L^−1^ in ethanol containing 0.4 mL L^−1^ collidine) and hung to dry for ~15 min, revealing anions (yellow) and cations (blue). After drying, the same papers were stained for sugars with AgNO_3_ (Fry, 2000).

For gel-permeation chromatography, as described by Iqbal *et al.* (2016), a 5.5 mL sample of concentrated LCSE was applied to a 185-mL bed-volume column of Bio-Gel P-2 and eluted in deionized water. The fractions collected were bioassayed on amaranth seedlings, and small samples were analysed by TLC.

TLC was performed in silica-gel plates run in BAW (4:1:1 by vol.), and sugars were stained with thymol/H_2_SO_4_ (Jork *et al.*, 1994).

### Phase partitioning of LCSE active principles

To test the effect of pH on the hydrophobicity of LCSE active principles, we dried 1.33 mL of LCSE (solid content 20 mg mL^−1^) as an 8 cm × 1 cm streak on Whatman No. 3 paper. Replicate papers were then wetted with a volatile buffer (formic acid/acetic acid/H_2_O, 1:4:45, pH 2.0; pyridine/acetic acid/H_2_O, 33:1:300, pH 6.5; or dilute ammonia, pH 9.0) and dried in a stream of air or shaken in 100 mL butan-1-ol for 30 min, then dried, or shaken in 100 mL toluene for 30 min, then dried. All papers were then thoroughly dried, and any LCSE that remained on the paper was eluted with water, repeatedly freeze-dried and redissolved in water and, finally, redissolved in 6.65 mL of water. This solution, which would have a concentration of 4 mg mL^−1^ (w/v) LCSE if no solutes had been lost into the organic solvents or remained bound to the paper, was bioassayed on amaranth seedlings. Control seedlings received pure water or never-dried LCSE.

### Statistics and graphical representation

Data analysis was performed with the computer software Statistix for the ANOVA at a significance level of *P* = 0.05, usually better. For graphical representation, data from MS Excel files were transferred to SigmaPlot v.14.0 to produce the histograms presented here.

## RESULTS

### Confirming the existence and basic properties of root-growth inhibitor

Amaranth seeds were incubated in the presence of various numbers of cress seeds. The presence of cress seeds had no effect on amaranth seed germination but had two opposing effects on seedling growth (Fig. 1). The elongation of the amaranth hypocotyls was strongly promoted. The hypocotyl-promoting principle, previously suggested to be lepidimoic acid (Hasegawa *et al.*, 1992), has more recently been shown to be potassium ions (Fry, 2017). The largest number of cress seeds tested (12) slightly diminished the promotion of amaranth hypocotyl elongation, probably because the elongation-promoting effect of K^+^ was countered by other products excreted by the cress; however, this effect was not statistically significant (*P* ≈ 0.1).

The elongation of the amaranth roots was strongly inhibited by the presence of cress (Fig. 1). The nature of the presumed root-inhibiting allelochemical remained unknown and was therefore investigated in the present paper.

*A priori*, an effect of neighbouring cress seed(ling)s could be attributable to competition with the amaranth seedlings for an essential resource, such as O_2_ (in a sealed Petri dish), or it could be attributable to the secretion of cress allelochemical(s). The latter interpretation was supported by experiments in which we collected LCSE and applied this to amaranth in the absence of live cress (Fig. 2). This experiment clearly demonstrated a strong promoting effect of LCSE on amaranth hypocotyl growth (previously shown to be attributable to the presence of K^+^; Fry, 2017) and a strong inhibitory effect on amaranth root growth.

The measured osmolality of LCSE was ~10.9 mOsmol kg^−1^ (Supplementary Data Table S1), equivalent to ~11 mm of total osmotically active molecules and ions. This compares closely to the measured total solute concentration of LCSE, 1.6 mg mL^−1^, which, if attributable entirely to hexoses, would correspond to ~9 mm (contributing ~0.022 MPa to the osmotic pressure). To investigate the possibility that osmotic pressure was the cause of the observed inhibition of root growth (by opposing water uptake for cell expansion), we tested the effect of 0–64 mm glucose or KCl on amaranth seedlings (measured osmolality, 0–129 mOsmol kg^−1^). Glucose in this concentration range had no appreciable effect on seedling growth (either roots or hypocotyls; Supplementary Data Table S1). KCl and a mixture containing glucose and KCl also had a negligible effect on root growth but promoted hypocotyl elongation, as expected for these concentrations of K^+^. Thus, osmotic pressure is unlikely to be the cause of root growth inhibition effected by LCSE.

To explore some fundamental properties of the root growth inhibitor, we subjected LCSE to various treatments and then re-tested its effect on amaranth seedlings (Fig. 2). Neither the hypocotyl promoter nor the root inhibitor was lost after drying or after incubation with hot or cold formic acid. Hot formic acid would have hydrolysed any lepidimoic acid, which was thus not the active principle. Both the hypocotyl promoter and the root inhibitor were found to partition into water, not ethyl acetate, indicating that the active principle was hydrophilic. The major difference was that the root inhibitor was lost upon ashing in a Bunsen flame, indicating that it was organic, whereas the hypocotyl promoter activity was retained. Thus, the root inhibitor comprises a heat-stable, hydrophilic, combustible compound(s) (thus not K^+^), whose bioactivity is retained in conditions that would hydrolyse disaccharides, including lepidimoic acid.

### Partitioning of LCSE active principles into organic solvents

We investigated the hydrophobicity of the active principles of LCSE at various pH values, which might affect their ionization if carboxy or amino groups are present. LCSE solutions buffered at pH 2.0, 6.5 or 9.0, applied to chromatography paper, were shaken with butanol or toluene or kept in the absence of partitioning solvents. Neither the hypocotyl promoter nor the root inhibitor was lost from the damp paper at any pH (Supplementary Data Fig. S1). There appeared to be partial loss of both active principles into butanol from pH 9 solution. The main conclusion is that both active principles are hydrophilic at all three pH values and are therefore unlikely to be lipophilic weak acids (e.g. abscisic acid) or lipophilic weak bases (e.g. sphingenine).

### Tolerance of amaranth seedlings to residues left after drying commonly used solvents

A promising approach for analysis of the active principles of LCSE would be chromatography or electrophoresis followed by bioassay of the separated fractions. Preliminary work suggested that some solvents commonly used during chromatography and electrophoresis might inhibit amaranth seedling growth. We therefore tested several solvents likely to be used (Supplementary Data Fig. S2). Compared with pure water, the residues obtained after drying of two volatile electrophoresis buffers (pH 2.0 and 6.5), the coolant used for electrophoresis at pH 6.5 (toluene) and the paper chromatography solvent BAW all had little or no effect on amaranth germination and seedling growth. However, the coolant used for electrophoresis at pH 2.0 (white spirit; ‘turpentine substitute’) completely blocked germination (Supplementary Data Fig. S2). The toxic constituent of white spirit could not be removed by repeated drying from alternative solvents, such as toluene, acetone or methanol (data not shown). Thus, electrophoresis at pH 2.0 was not compatible with bioassays and was not explored further in this work. However, electrophoresis at pH 6.5 and chromatography in BAW were suitable methods for separation.

### Deleterious effects of certain salts on root growth

As expected, all tested K^+^ salts (at 5 or 10 mm) were able to promote amaranth hypocotyl elongation; Na^+^ salts did not have this effect (Fig. 3). We investigated whether salts might be root growth inhibitors, because such an effect would complicate future attempts to analyse the natural allelochemical(s) present in LCSE. Indeed, many of the tested salts tended to inhibit root growth, especially 10 mm salts of organic anions (Fig. 3A–C); however, phosphate and the chlorides were the least inhibitory. These observations raise the possibility that LCSE fractions eluted from paper chromatograms or electrophoretograms that had been run in the presence of acetic or formic acid might inhibit root growth owing to the acetate or formate trapped by naturally occurring cations (K^+^ being the most abundant cation in LCSE). Thus, added acetic or formic acid might convert endogenous K^+^ into an apparent ‘root inhibitory principle’ of no biological significance.

Hypocotyl growth was promoted by the acetate, formate and chloride salts of K^+^, but less so by the trifluoroacetate salt; root growth was inhibited by all the K^+^ salts tested except the chloride (Fig. 3C). Thus, after chromatography or electrophoresis of LCSE specimens, it would be important to remove any formate, acetate or trifluoroacetate carried over from the solvents and which might otherwise be trapped in the sample by the K^+^ and other cations present in LCSE.

We tested whether the root-inhibiting effect of potassium acetate might be alleviated if we dried the sample from an excess of an alternative volatile acid, such as HCl, formic acid or trifluoroacetic acid (TFA), whose anions might be less toxic. After drying, such samples would become predominantly KCl, potassium formate and potassium TFA, respectively. Indeed, potassium acetate whose acetate had been largely removed by drying from HCl or TFA was less inhibitory to root growth than equimolar potassium acetate (Fig. 3D). Formic acid was not useful in this way. Thus, an excess of HCl could potentially be used to drive off any trapped acetate or formate from K^+^-containing fractions eluted from chromatograms and electrophoretograms. TFA is less suitable in this capacity because it interferes with the stimulatory effect of K^+^ on hypocotyl growth (Fig. 3C, D).

The above data suggest that, although K^+^ is not the principal root inhibitor, it might interfere in root bioassays by forming non-volatile, growth-inhibitory salts when insufficiently dried from experimental samples, such as eluates from chromatograms and electrophoretograms.

### Electrophoresis of LCSE at pH 6.5

Given that the bioactive principles do not partition into toluene and that the buffers and toluene used can be removed by thorough drying, we characterized the root-inhibiting principle of LCSE by high-voltage electrophoresis at pH 6.5. LCSE contained abundant neutral sugars (not stained by Bromophenol Blue but detected by sugar stains; results not shown) and several ions, including K^+^, galacturonic acid, phosphate, sulphate and an unidentified anion (Fig. 4A). Root inhibitory activity was observed in electrophoretogram zone 6, which is where neutral and slow-migrating cationic solutes run. As expected, the hypocotyl promoter ran in the region of K^+^. The results are compatible with the root inhibitor possessing no net charge at pH 6.5, thus possibly a neutral sugar. Neutral sugars, of course, do not separate from each other on electrophoresis and cannot be identified by this method.

### Paper chromatography of LCSE

To gain further insight into the nature of the active principle(s), we fractionated LCSE by paper chromatography in butanol/acetic acid/water (12:3:5) and bioassayed zones of the chromatograms after thorough removal of the solvents. The mobilities of various relevant solutes in this chromatography system are shown in Supplementary Data Fig. S3. In Supplementary Data Fig. S3A, the ionic components are stained with Bromothymol Blue (anions stain yellow, cations blue or violet–orange in the case of Zn^2+^ and Cu^2+^); in Supplementary Data Fig. S3B, the sugars are stained brownish with silver nitrate (some ions show as pale spots against the weakly stained cellulose of the paper). LCSE contained detectable K^+^, lepidimoic acid, galacturonic acid, glucose, fructose, sulphate, phosphate and an unidentified fast-migrating anion.

On separate chromatograms, 100-µL streak loadings of 15 independent LCSE samples were similarly run alongside marker mixtures and identical streaks of the same 15 LCSE samples. As shown in Fig. 5 for five examples of the 15, the markers and one set of LCSE loadings were stained with Bromophenol Blue, revealing the ions present (Fig. 5A), followed by silver nitrate, revealing the sugars (Fig. 5B). Fractions (‘zones 1–10’) from the unstained LCSE run were then bioassayed for effects on amaranth seedling growth (Fig. 5C, D). The hypocotyl growth promoter was found (zones 4 > 5), corresponding to K^+^, as expected. No significant inhibition of amaranth root growth was observed in any zone. It is possible that two or more components are required for detectable root inhibition and that they were separated by the chromatography.

### Gel-permeation chromatography of LCSE

Gel-permeation chromatography on Bio-Gel P-2 separates compounds principally on the basis of size and within the range ~100–2000 kDa. The constituents of LCSE were resolved into soluble polysaccharides in fractions 16–24, then a series of oligo- and monosaccharides, some of which can be identified provisionally on the TLC (glucose + fructose, peaking in fractions 36–42; sucrose in fractions 34–40; galacturonic acid in fractions 36–42; and a putative trisaccharide in fractions 28–36) (Fig. 6). Indeed, trisaccharides, such as raffinose, have recently been reported to be present in LCSE (Lijina *et al.*, 2023). In addition, an unidentified fast-migrating sugar was observed in Bio-Gel fractions 48–50, previously referred to as B1 and shown to be a non-reducing, acidic disaccharide (fig. 5 in the paper by Iqbal *et al.*, 2016). The peak of root inhibition (*P* < 0.001) was in fraction 38, coinciding with the monosaccharides; several neighbouring fractions also tended to inhibit root growth, albeit without statistical significance. The strong peak of hypocotyl promotion in fractions 30–38 was previously reported to be attributable to K^+^ (Fry, 2017). Two further repeats of this experiment on a smaller scale confirmed all the above trends, with strong statistical significance (Supplementary Data Fig. S4). The results indicate that inhibition of root growth was attributable to small, water-soluble solute(s), of approximately the size of monosaccharides and apparently smaller than disaccharides (which would include lepidimoic acid).

## DISCUSSION

Considerable interest centres on allelochemicals from the points of view of basic plant physiology, applications in agriculture, including ‘green’ herbicide development, and ecophysiology. Clearly, plants compete in the natural environment, and allelochemicals help to give certain species a selective advantage over their neighbours. We have focused on the ability of cress seeds to exude substances that adversely affect the growth of neighbouring ‘competitors’ [in our model experiments, amaranth seedlings to facilitate comparison with much of the earlier work (Hasegawa *et al.*, 1992; Iqbal *et al.*, 2012)].

It was previously established that K^+^, exuded from cress seeds as a component of LCSE, acts to over-stimulate the elongation of amaranth hypocotyls and restrict their growth in girth, thus weakening them (Hasegawa *et al.*, 1992; Iqbal *et al.*, 2012). In our present work, the elongation of the amaranth hypocotyls was strongly promoted by LCSE.

Conversely, the elongation of the amaranth roots was strongly inhibited by the presence of cress seed(ling)s (Fig. 1), a highly reproducible adverse effect also initially suggested to be attributable to the presence of lepidimoic acid, a rhamnogalacturonan-I-derived disaccharide (Hasegawa *et al.*, 1992). However, more recent work indicated that neither lepidimoic acid nor K^+^ was responsible for root growth inhibition (Fry, 2017). Indeed, later work from Hasegawa’s group did not strongly support lepidimoic acid (Yamada *et al.*, 2007). The root inhibitors are organic and clearly not K^+^ ions; and KCl at reasonable concentrations did not affect root growth in the present work. However, the nature of the root growth inhibitor was unknown and has been investigated here.

The present paper confirms that cell-free LCSE mimics the presence of live cress seed(ling)s and robustly inhibits amaranth root growth (Fig. 2B) and shows that the inhibitor responsible is non-volatile (not lost on drying), organic (destroyed on ashing, unlike K^+^), strongly hydrophilic (thus not one of the classic phytohormones) and stable to hot acid (unlike a disaccharide). The main aim of the present paper was to characterize the root growth inhibitor(s) present in cress-seed exudate. Our working hypothesis, developed below, is that the root inhibitory principle is a mixture of small molecules.

Before a more detailed study of the root inhibitor(s) present in LCSE, we needed to overcome certain practical difficulties, and the findings of this part of the work will assist future studies on the active principles present in LCSE and any comparable allelochemical preparations. We tested the phytotoxicity of residues left after evaporating several solvents commonly used for chromatography and electrophoresis (Supplementary Data Fig. S2) and found that all were acceptable except white spirit (‘turpentine substitute’), which is routinely used as the coolant during paper electrophoresis in pH 2.0 buffer (Fry, 2020). Even after repeated drying of white spirit, including re-drying from any of a range of other solvents, an (invisible and odourless) substance evidently remained, which completely inhibited amaranth seed germination. Thus, the active principle(s) of LCSE could not be studied by electrophoresis at pH 2.0. Nevertheless, the pH 2.0 solvent itself, in addition to the coolant (toluene) and buffers for electrophoresis at pH 6.5, and the paper chromatography solvent BAW, were satisfactory.

Concerning another potential artefact, we also found that K^+^, the major cation present in LCSE, although not itself inhibiting root growth when added as KCl, KH_2_PO_4_ or K_2_SO_4_, was capable of trapping otherwise volatile acids (acetic and formic) that would often be added during chromatography or electrophoresis (Fig. 3). To overcome this problem, we found that it was possible to exchange the acetate or formate for chloride by adding a small excess of HCl, then drying it off (Fig. 3D). The great majority of the acetate and formate would then be released as volatile acetic and formic acids; the excess HCl would also be volatilized, and the non-volatile K^+^ would be left as innocuous KCl.

Taking precautions to overcome the above potential problems, we were able to gain further insight into the nature of the root inhibitor(s) present in LCSE. They remained in the aqueous phase (at all pH values tested) when partitioned against butan-1-ol or toluene and were thus highly hydrophilic. Most naturally occurring carboxylic acids lose their negative charge at pH 2, which would enhance their ability to partition into butanol. Likewise, most naturally occurring amino compounds lose their positive charge at high pH, favouring their partitioning into butanol (such as, for example, sphingosine). The inability of the root inhibitors to partition into organic solvents from water at any pH suggests that they were highly hydrophilic (e.g. sugars) and not hydrophobic weak acids or bases.

To investigate the ionization of the root inhibitors, we performed high-voltage electrophoresis of LCSE and bioassayed the fractions. In these conditions, the hypocotyl promoter gave a very prominent peak of bioactivity co-migrating with K^+^ (Fig. 4A), as expected. However, the root inhibitor, which was at first highly bioactive (Fig. 2B), gave only a weak zone of bioactivity on the electrophoretogram (Fig. 4B). The minority of the root-inhibitory principle that did remain statistically detectable was in the neutral region (zone 6), co-migrating with glucose and fructose (Fig. 4C). The loss of some of the activity, despite the fact that the root inhibitor did not partition into toluene (used as a coolant for electrophoresis at pH 6.5), suggests that the root inhibitor comprised multiple compounds, which would synergize but were resolved, in part, by electrophoresis. Indeed, there was a hint of root-inhibitory activity in zones 3 and 8, albeit not strongly different (*P* > 0.01) from the mean of the four zones that permitted maximum root elongation (Fig. 4C). The idea of multiple components was supported by the total loss of the detectable root inhibitory activity of LCSE upon paper chromatography (Fig. 5D), which is considered to have resolved multiple different solutes such that they were unable to act synergistically.

On gel-permeation (size-exclusion) chromatography on Bio-Gel P-2, the root inhibitor co-eluted with neutral hexoses (molecular weight, 180), indicating that most of the root-inhibiting constituents of LCSE were small molecules.

In summary, although the specific components of cress-seed exudate that interfere with the root growth of neighbouring competitor seedlings remain to be identified, they are concluded to be a mixture of small organic molecules, some of them neutral (migrating in zone 6 of the electrophoretogram) and others charged [thus distributed over other zone(s) of the electrophoretogram and therefore unable to contribute synergistically to an allelopathic effect; Fig. 4], all of which would need to be present in order to exert the full biological impact on competitor seedlings. Such a mixture of small molecules might *a priori* be suggested to have its effect on root growth by building up a sufficiently high osmotic pressure (which translates into a negative water potential in the surrounding medium) to suppress water uptake and thus root cell expansion. However, this hypothesis was rejected because: (1) the measured osmolality of LCSE was only 11 mOsmol kg^−1^ (equivalent to 11 mm of total sugars); and (2) the total concentration of solutes in LCSE was only ~1.6 mg mL^−1^, which, if attributable to hexose monosaccharides, would equate to ~9 mm, a concentration at which glucose had no discernible effect on root growth (Supplementary Data Table S1).

The allelochemicals found here to be exuded by cress seeds clearly inhibit root growth in neighbouring amaranth plants. Our work focused on an arbitrarily selected model system: cress vs. amaranth. We do not expect this pair of plant species to occur frequently in close proximity; however, the cress-seed allelochemicals documented here might well inhibit root growth of many other species that do tend to compete for establishment in the same soil, either horticulturally or in the wild. In addition, the seeds of numerous other plant species probably also exude comparable allelochemicals. There remain many exciting opportunities to detect and characterize new allelochemicals by experiments similar to those used here.

### Conclusions

Cress seeds exude a range of substances that together act as allelochemicals, interfering with the growth of neighbouring (potentially competing) seedlings (amaranth, in our model experimental system). These low-molecular weight substances include K^+^ ions, which specifically over-stimulate hypocotyl elongation, plus a cocktail of small, hydrophilic, heat-stable, organic compounds (including, as identified by chromatography and electrophoresis, glucose, fructose, sucrose and galacturonic acid) that together inhibit root growth. These major sugars of cress-seed exudate, however, are present at too low a total concentration (even if augmented by K^+^) to inhibit root growth by creating a high osmotic pressure. Thus, the root-inhibitory allelopathic principle is not a single specific allelochemical, but rather a mixture of small organic molecules which, however, remain to be identified.

## SUPPLEMENTARY DATA

Supplementary data are available at *Annals of Botany* online and consist of the following.

Figure S1: failure of low-molecular-weight cress-seed exudate (LCSE) active principles to partition from water into immiscible organic solvents. Figure S2: effect of residues from commonly used solvents on amaranth seedling growth. Figure S3: paper chromatography of low-molecular-weight cress-seed exudate (LCSE) and some relevant markers. Figure S4: two replicate studies of the behaviour of the active principles of low-molecular-weight cress-seed exudate (LCSE) on gel-permeation chromatography. Table S1: Effects of osmotica on amaranth seedling growth.

mcad200_suppl_Supplementary_Material

## References

[CIT0001] Asaduzzaman M , An M, Pratley JE, Luckett DJ, Lemerle D, Coombes N. 2016. The seedling root response of annual ryegrass (*Lolium rigidum*) to neighbouring seedlings of a highly-allelopathic canola (*Brassica napus*). Flora 219: 18–24.

[CIT0002] Boydston RA , Morra MJ, Borek V, Clayton L, Vaughn SF. 2011. Onion and weed response to mustard (*Sinapis alba*) seed meal. Weed Science 59: 546–552.

[CIT0003] Cordeau S , Triolet M, Wayman S, Steinberg C, Guillemin J-P. 2016. Bioherbicides: dead in the water? A review of the existing products for integrated weed management. Crop Protection 87: 44–49.

[CIT0004] Cornes D. 2005. Callisto: a very successful maize herbicide inspired by allelochemistry. Proceedings of 4th World Congress on Allelopathy, Wagga Wagga, NSW, Australia. http://www.regional.org.au/au/allelopathy/2005/2/7/2636_cornesd.htm

[CIT0005] Curl EA , Truelove B. 1986. The rhizosphere. New York: Springer.

[CIT0006] Dayan FE , Howell J, Marais JP, Ferreira D, Koivunen M. 2011. Manuka oil, a natural herbicide with preemergence activity. Weed Science 59: 464–469.

[CIT0007] Fan TW-M , Lane AM, Crowley D, Higashi RM. 1997. Comprehensive analysis of organic ligands in whole root exudate using nuclear magnetic resonance and gas chromatography–mass spectrometry. Analytical Biochemistry 251: 57–68. 9300083 10.1006/abio.1997.2235

[CIT0008] Fry SC. 2000. The growing plant cell wall: chemical and metabolic analysis. Reprint edn. Caldwell : The Blackburn Press.

[CIT0009] Fry SC. 2017. Potassium, not lepidimoide, is the principal ‘allelochemical’ of cress-seed exudate that promotes amaranth hypocotyl elongation. Annals of Botany 120: 511–520. 28981578 10.1093/aob/mcx081 PMC5737857

[CIT0010] Fry SC. 2020. High-voltage paper electrophoresis (HVPE). In: Popper Z. ed. The plant cell wall. Methods in molecular biology, Vol. 2149. New York: Humana, 1–31. 10.1007/978-1-0716-0621-6_1 32617926

[CIT0011] Hale MG , Moore LD, Griffin GJ. 1978. Root exudate and exudation. In: Domergues VR, Krupa SV. eds. Interactions between non-pathogenic soil microorganisms and plants. Amsterdam: Elsevier, 163–203.

[CIT0012] Hasegawa K , Mizutani J, Kosemura S, Yamamura S. 1992. Isolation and identification of lepidimoide, a new allelopathic substance from mucilage of germinated cress seeds. Plant Physiology 100: 1059–1061. 16653018 10.1104/pp.100.2.1059 PMC1075667

[CIT0013] He Z , Yao L, Zhang X, Li Y, Guo YJ. 2019. Faba bean organs differed in their effects on maize seed germination rate and soil microbial activities as well as their decomposition patterns in a regosol soil. Journal of Soil Science and Plant Nutrition 20: 367–379.

[CIT0014] Higashinakasu K , Yamada K, Shigemori H, Hasegawa K. 2004. Effects of seed exudates of several plant species during germination stage. Weed Biology and Management 4: 171–175.

[CIT0015] Iqbal A , Fry SC. 2012. Potent endogenous allelopathic compounds in *Lepidium sativum* seed exudate: effects on epidermal cell growth in *Amaranthus caudatus* seedlings. Journal of Experimental Botany 63: 2595–2604. 22268144 10.1093/jxb/err436 PMC3346223

[CIT0016] Iqbal A , Miller JG, Murray L, Sadler IH, Fry SC. 2016. The pectic disaccharides lepidimoic acid and β-d-xylopyranosyl-(1→3)-d-galacturonic acid occur in cress-seed exudate but lack allelochemical activity. Annals of Botany 117: 607–623. 26957370 10.1093/aob/mcw008 PMC4817500

[CIT0017] Jassbi AR , Zamanizadehnajari S, Baldwin IT. 2010. Phytotoxic volatiles in the roots and shoots of *Artemisia tridentata* as detected by headspace solid-phase microextraction and gas chromatographic–mass spectrometry analysis. Journal of Chemical Ecology 36: 1398–1407. 21086024 10.1007/s10886-010-9885-0

[CIT0018] Jork H , Funk W, Fischer W, Wimmer H. 1994. *Thin-layer chromatography: reagents and detection methods*, Vol. 1b: *physical and chemical detection methods; activation reactions, reagents sequences, reagents II*. Weinheim: VCH.

[CIT0019] Khan I , Khan MI. 2015. Environment friendly (allelopathic extract) weed control techniques in wheat crop. Revista Mexicana de Ciencias Agrícolas 6: 1306–1316.

[CIT0020] Kong C-H , Zhang S-Z, Li Y-H, Xia Z-C, Meiners SJ, Wang P. 2018. Plant neighbor detection and allelochemical response are driven by root-secreted signaling chemicals. Nature Communications 9: 3867. 10.1038/s41467-018-06429-1 PMC6155373 30250243

[CIT0021] Lee KC , Campbell RW. 1969. Nature and occurrence of juglone in *Juglans nigra* L. HortScience 4: 297–298.

[CIT0022] Li LL , Zhao HH, Kong CH. 2020. Loliolide, the most ubiquitous lactone, is involved in barnyard grass-induced rice allelopathy. Journal of Experimental Botany 71: 1540–1550. 31677347 10.1093/jxb/erz497

[CIT0023] Li YH , Xia ZC, Kong CH. 2016. Allelobiosis in the interference of allelopathic wheat with weeds. Pest Management Science 72: 2146–2153. 26833449 10.1002/ps.4246

[CIT0024] Lijina P , Manjunatha JR, Kumar BSG. 2023. Characterization of free oligosaccharides from garden cress seed aqueous exudate using PGC LC-MS/MS and NMR spectroscopy. Carbohydrate Research 532: 108914. 37541111 10.1016/j.carres.2023.108914

[CIT0025] Long S , Lendzemo V, Kuyper TW, Kang Z, Vierheilig H, Steinkellner S. 2008. A simple staining method for observation of germinated *Striga* seeds. Seed Science Research 18: 125–129.

[CIT0026] McIntyre GI , Boyer JS. 1984. The effect of humidity, root excision, and potassium supply on hypocotyl elongation in dark-grown seedlings of *Helianthus annuus*. Canadian Journal of Botany 62: 420–428.

[CIT0027] Molisch H. 1937. Der Einfluss einer Pflanze auf die Andere, Allelopathie. Jena: Gustav Fischer.

[CIT0028] Rice EL. 1984. Allelopathy, 2nd edn. Orlando: Academic Press, Incharge.

[CIT0029] Rosko J , Martinez VA, Poon WCK, Pilizota T. 2017. Osmotaxis in *Escherichia coli* through changes in motor speed. Proceedings of the National Academy of Sciences of the United States of America 114: E7969–E7976. 28874571 10.1073/pnas.1620945114 PMC5617246

[CIT0030] Rovira AD. 1969. Plant root exudates. Botanical Review 35: 35–57.

[CIT0031] Sahu A , Devkota A. 2013. Allelopathic effects of aqueous extract of leaves of *Mikania micrantha* H.B.K. on seed germination and seedling growth of *Oryza sativa* L. and *Raphanus sativus* L. Scientific World 11: 90–93.

[CIT0032] Sarkar E , Chatterjee SN, Chakraborty P. 2012. Allelopathic effect of *Cassia tora* on seed germination and growth of mustard. Turkish Journal of Botany 36: 488–494.

[CIT0033] Szwed M , Mitrus J, Wiczkowski W, Dębski H, Horbowicz M. 2020. If phenolic compounds in the soil with buckwheat residues affect the emergence and growth of weed seedlings? Acta Physiologiae Plantarum 42: 154.

[CIT0034] Uren NC. 2000. Types, amounts, and possible functions of compounds released into the rhizosphere by soil-grown plants. In: Pinton R, Varanini Z, Nannipieri P. eds. The rhizosphere: biochemistry and organic substances at the soil–plant interface. New York: Marcel Dekker, 19–40.

[CIT0035] Willis RJ. 2007. The history of allelopathy. Dordrecht, Netherlands: Springer.

[CIT0036] Yamada K , Miyamoto K, Goto N, et al. 2007. Chemical and biological analysis of novel allelopathic substances, lepidimoide and lepidimoic acid. In: Fujii Y, Hiradate S. eds. Allelopathy: new concepts and methodology. Enfield: Science Publisher, 123–135.

[CIT0037] Yang X-F , Kong C-H. 2017. Interference of allelopathic rice with paddy weeds at the root level. Plant Biology 19: 584–591. 28218979 10.1111/plb.12557

