# Supplementary material for: Allelochemical root-growth inhibitors in low-molecular-weight cress-seed exudate
Source: Ann Bot. 2023 Dec 23;133(3):447–58. doi: 10.1093/aob/mcad200 (PMC11006535; doi:10.1093/aob/mcad200)
Supplement: mcad200_suppl_Supplementary_Material [file mcad200_suppl_supplementary_material.pptx]

## Slide 1
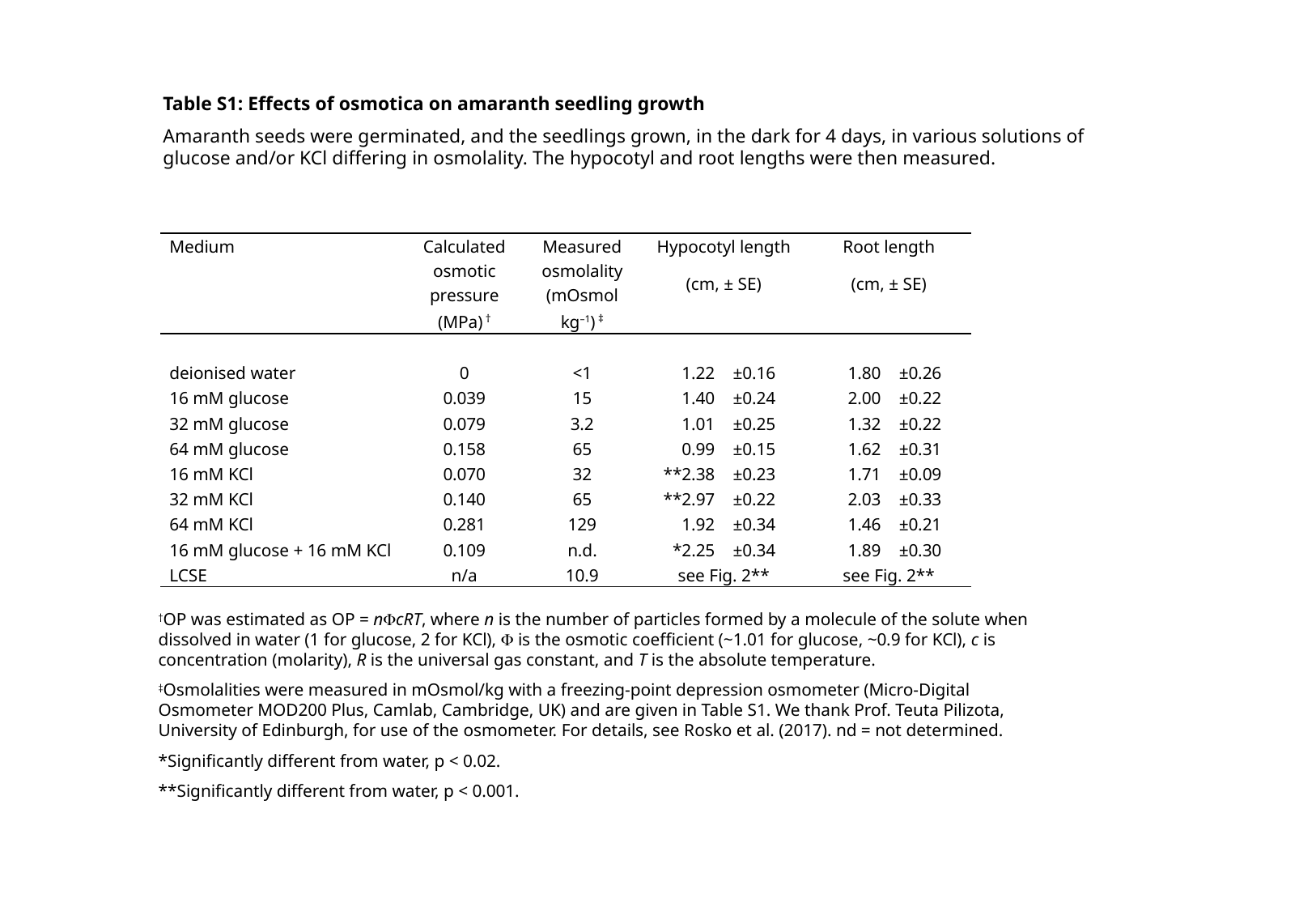

Table S1: Effects of osmotica on amaranth seedling growth
Amaranth seeds were germinated, and the seedlings grown, in the dark for 4 days, in various solutions of glucose and/or KCl differing in osmolality. The hypocotyl and root lengths were then measured.
| Medium | Calculated osmotic pressure (MPa) † | Measured osmolality (mOsmol kg–1) ‡ | Hypocotyl length (cm, ± SE) | | Root length (cm, ± SE) | |
| --- | --- | --- | --- | --- | --- | --- |
| | | | | | | |
| deionised water | 0 | <1 | 1.22 | ±0.16 | 1.80 | ±0.26 |
| 16 mM glucose | 0.039 | 15 | 1.40 | ±0.24 | 2.00 | ±0.22 |
| 32 mM glucose | 0.079 | 3.2 | 1.01 | ±0.25 | 1.32 | ±0.22 |
| 64 mM glucose | 0.158 | 65 | 0.99 | ±0.15 | 1.62 | ±0.31 |
| 16 mM KCl | 0.070 | 32 | \*\*2.38 | ±0.23 | 1.71 | ±0.09 |
| 32 mM KCl | 0.140 | 65 | \*\*2.97 | ±0.22 | 2.03 | ±0.33 |
| 64 mM KCl | 0.281 | 129 | 1.92 | ±0.34 | 1.46 | ±0.21 |
| 16 mM glucose + 16 mM KCl | 0.109 | n.d. | \*2.25 | ±0.34 | 1.89 | ±0.30 |
| LCSE | n/a | 10.9 | see Fig. 2\*\* | | see Fig. 2\*\* | |
†OP was estimated as OP = ncRT, where n is the number of particles formed by a molecule of the solute when dissolved in water (1 for glucose, 2 for KCl),  is the osmotic coefficient (~1.01 for glucose, ~0.9 for KCl), c is concentration (molarity), R is the universal gas constant, and T is the absolute temperature.
‡Osmolalities were measured in mOsmol/kg with a freezing-point depression osmometer (Micro-Digital Osmometer MOD200 Plus, Camlab, Cambridge, UK) and are given in Table S1. We thank Prof. Teuta Pilizota, University of Edinburgh, for use of the osmometer. For details, see Rosko et al. (2017). nd = not determined.
*Significantly different from water, p < 0.02.
**Significantly different from water, p < 0.001.

## Slide 2
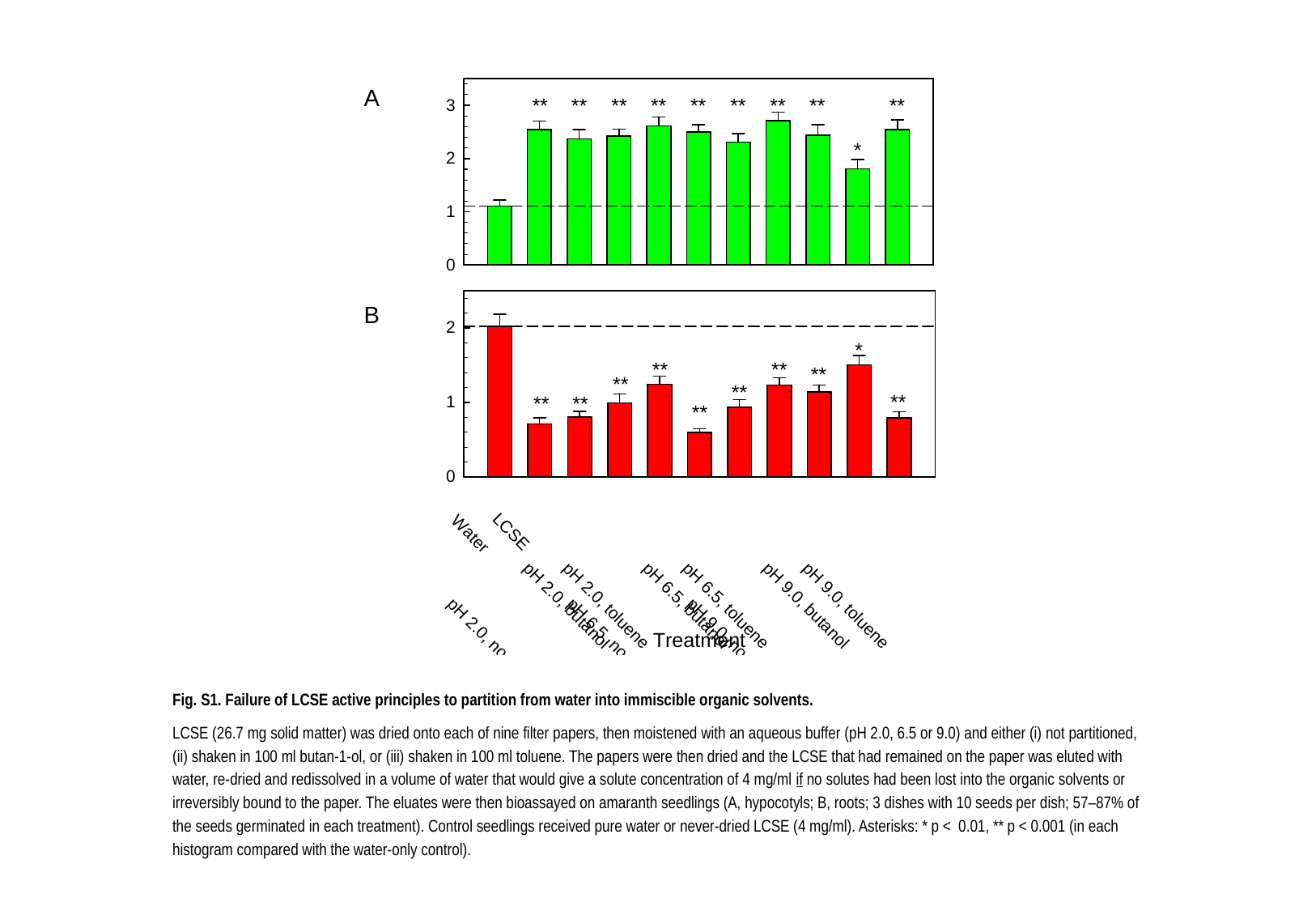

A
**
**
**
**
**
**
**
**
**
*
B
*
**
**
**
**
**
**
**
**
**
Fig. S1. Failure of LCSE active principles to partition from water into immiscible organic solvents.
LCSE (26.7 mg solid matter) was dried onto each of nine filter papers, then moistened with an aqueous buffer (pH 2.0, 6.5 or 9.0) and either (i) not partitioned, (ii) shaken in 100 ml butan-1-ol, or (iii) shaken in 100 ml toluene. The papers were then dried and the LCSE that had remained on the paper was eluted with water, re-dried and redissolved in a volume of water that would give a solute concentration of 4 mg/ml if no solutes had been lost into the organic solvents or irreversibly bound to the paper. The eluates were then bioassayed on amaranth seedlings (A, hypocotyls; B, roots; 3 dishes with 10 seeds per dish; 57–87% of the seeds germinated in each treatment). Control seedlings received pure water or never-dried LCSE (4 mg/ml). Asterisks: * p < 0.01, ** p < 0.001 (in each histogram compared with the water-only control).

## Slide 3
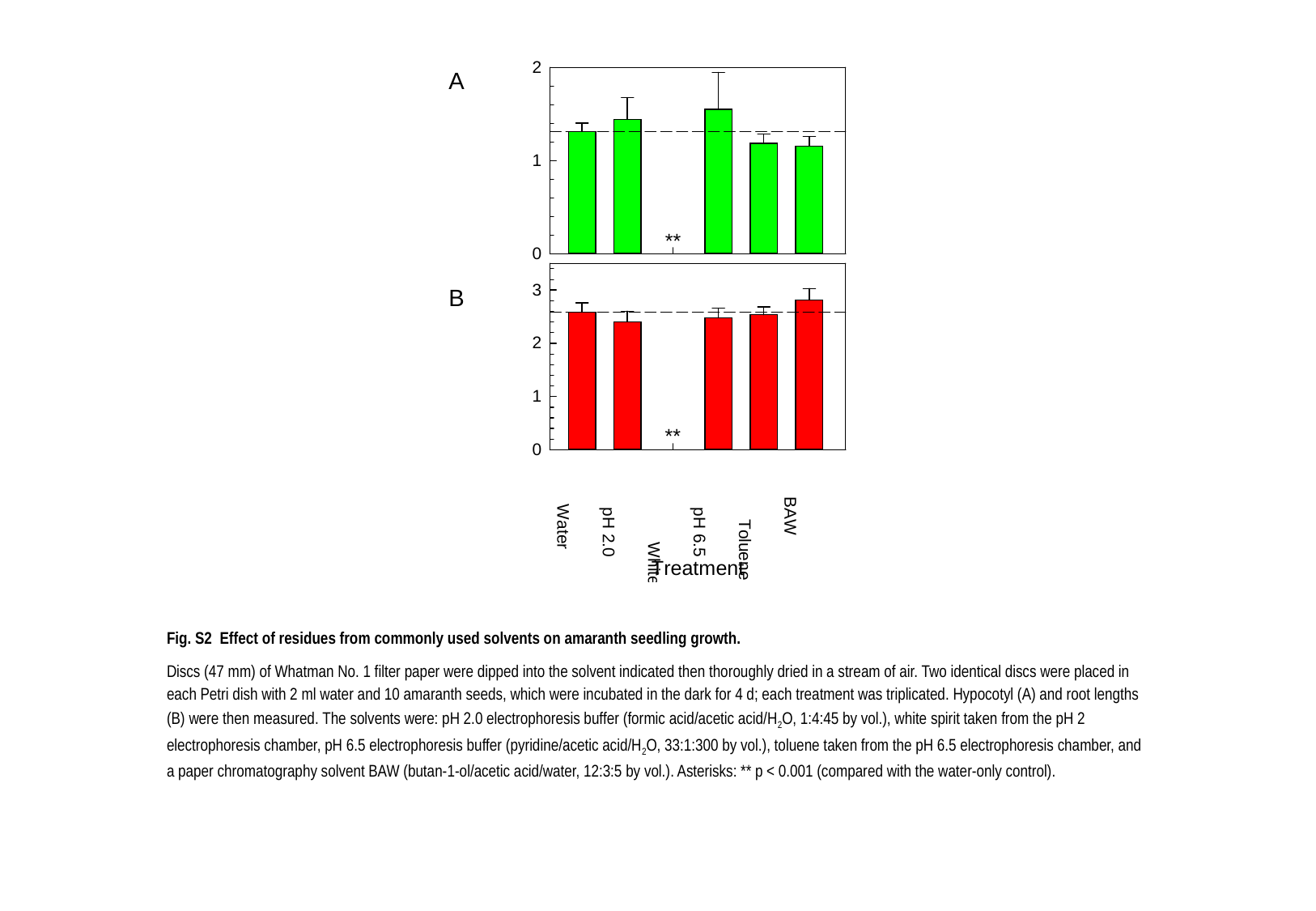

A
**
B
**
Fig. S2 Effect of residues from commonly used solvents on amaranth seedling growth.
Discs (47 mm) of Whatman No. 1 filter paper were dipped into the solvent indicated then thoroughly dried in a stream of air. Two identical discs were placed in each Petri dish with 2 ml water and 10 amaranth seeds, which were incubated in the dark for 4 d; each treatment was triplicated. Hypocotyl (A) and root lengths (B) were then measured. The solvents were: pH 2.0 electrophoresis buffer (formic acid/acetic acid/H2O, 1:4:45 by vol.), white spirit taken from the pH 2 electrophoresis chamber, pH 6.5 electrophoresis buffer (pyridine/acetic acid/H2O, 33:1:300 by vol.), toluene taken from the pH 6.5 electrophoresis chamber, and a paper chromatography solvent BAW (butan-1-ol/acetic acid/water, 12:3:5 by vol.). Asterisks: ** p < 0.001 (compared with the water-only control).

## Slide 4
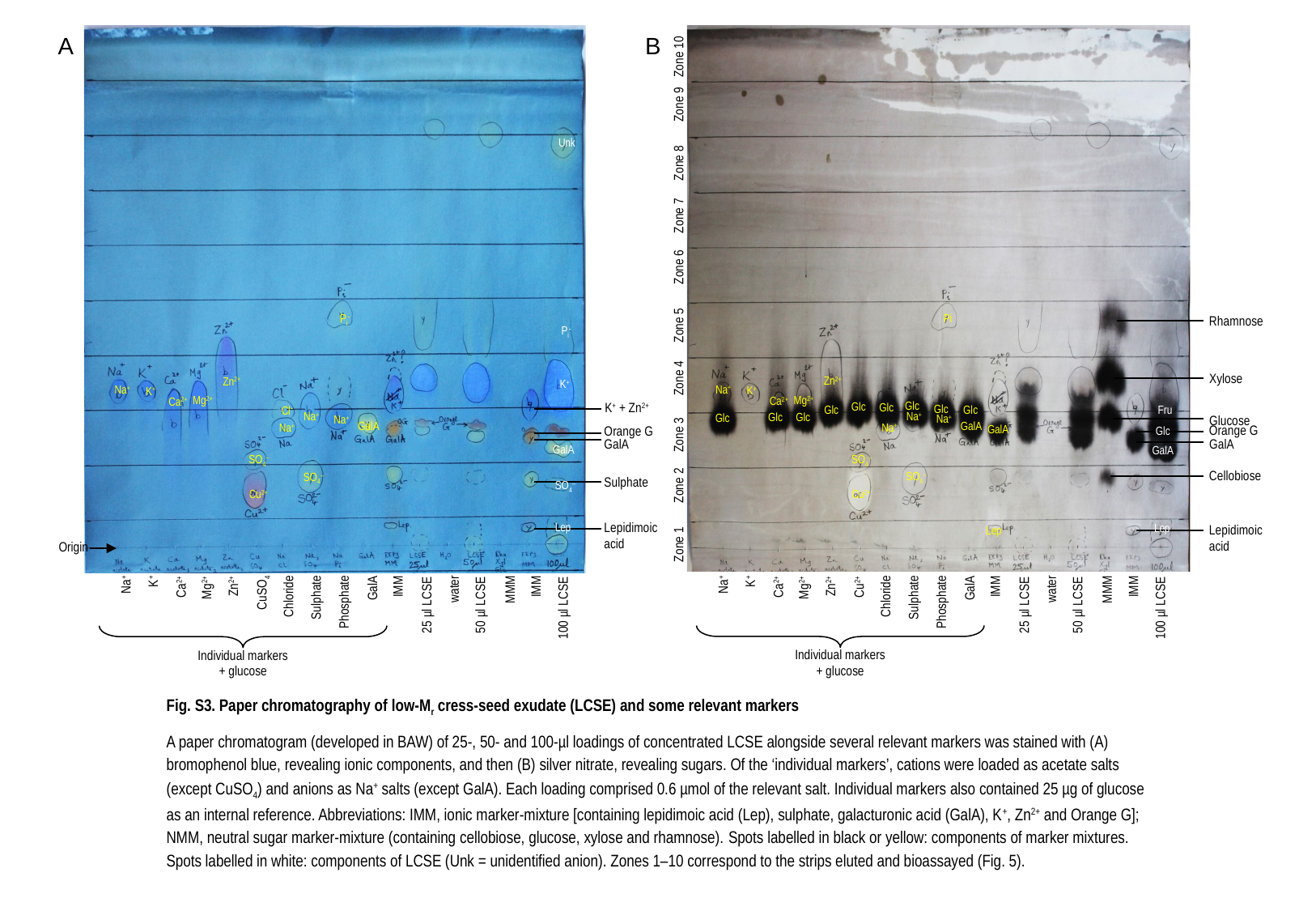

A
B
Zone 10
Zone 9
Unk
Zone 8
Zone 7
Zone 6
Pi
Pi−
Rhamnose
Zone 5
Pi−
Xylose
Zone 4
Zn2+
Zn2+
K+
Na+
Na+
K+
K+
Mg2+
Mg2+
Ca2+
Ca2+
Glc
K+ + Zn2+
Glc
Glc
Glc
Glc
Glc
Fru
Cl−
Na+
Na+
Glc
Glc
Glc
Na+
Glucose
Na+
GalA
GalA
Na+
Na+
Orange G
GalA
Orange G
Glc
Zone 3
GalA
GalA
GalA
GalA
SO4–
SO4–
Cellobiose
SO4–
SO4–
Sulphate
Zone 2
SO4–
Cu2+
Cu2+
Lepidimoic
acid
Lep
Lep
Lepidimoic
acid
Lep
Zone 1
Origin
K+
K+
Na+
Na+
Zn2+
Zn2+
IMM
IMM
IMM
IMM
Ca2+
Ca2+
Cu2+
Mg2+
Mg2+
GalA
GalA
water
water
MMM
MMM
CuSO4
Chloride
Chloride
Sulphate
Sulphate
Phosphate
Phosphate
25 µl LCSE
50 µl LCSE
25 µl LCSE
50 µl LCSE
100 µl LCSE
100 µl LCSE
Individual markers
+ glucose
Individual markers
+ glucose
Fig. S3. Paper chromatography of low-Mr cress-seed exudate (LCSE) and some relevant markers
A paper chromatogram (developed in BAW) of 25-, 50- and 100-µl loadings of concentrated LCSE alongside several relevant markers was stained with (A) bromophenol blue, revealing ionic components, and then (B) silver nitrate, revealing sugars. Of the ‘individual markers’, cations were loaded as acetate salts (except CuSO4) and anions as Na+ salts (except GalA). Each loading comprised 0.6 µmol of the relevant salt. Individual markers also contained 25 µg of glucose as an internal reference. Abbreviations: IMM, ionic marker-mixture [containing lepidimoic acid (Lep), sulphate, galacturonic acid (GalA), K+, Zn2+ and Orange G]; NMM, neutral sugar marker-mixture (containing cellobiose, glucose, xylose and rhamnose). Spots labelled in black or yellow: components of marker mixtures. Spots labelled in white: components of LCSE (Unk = unidentified anion). Zones 1–10 correspond to the strips eluted and bioassayed (Fig. 5).

## Slide 5
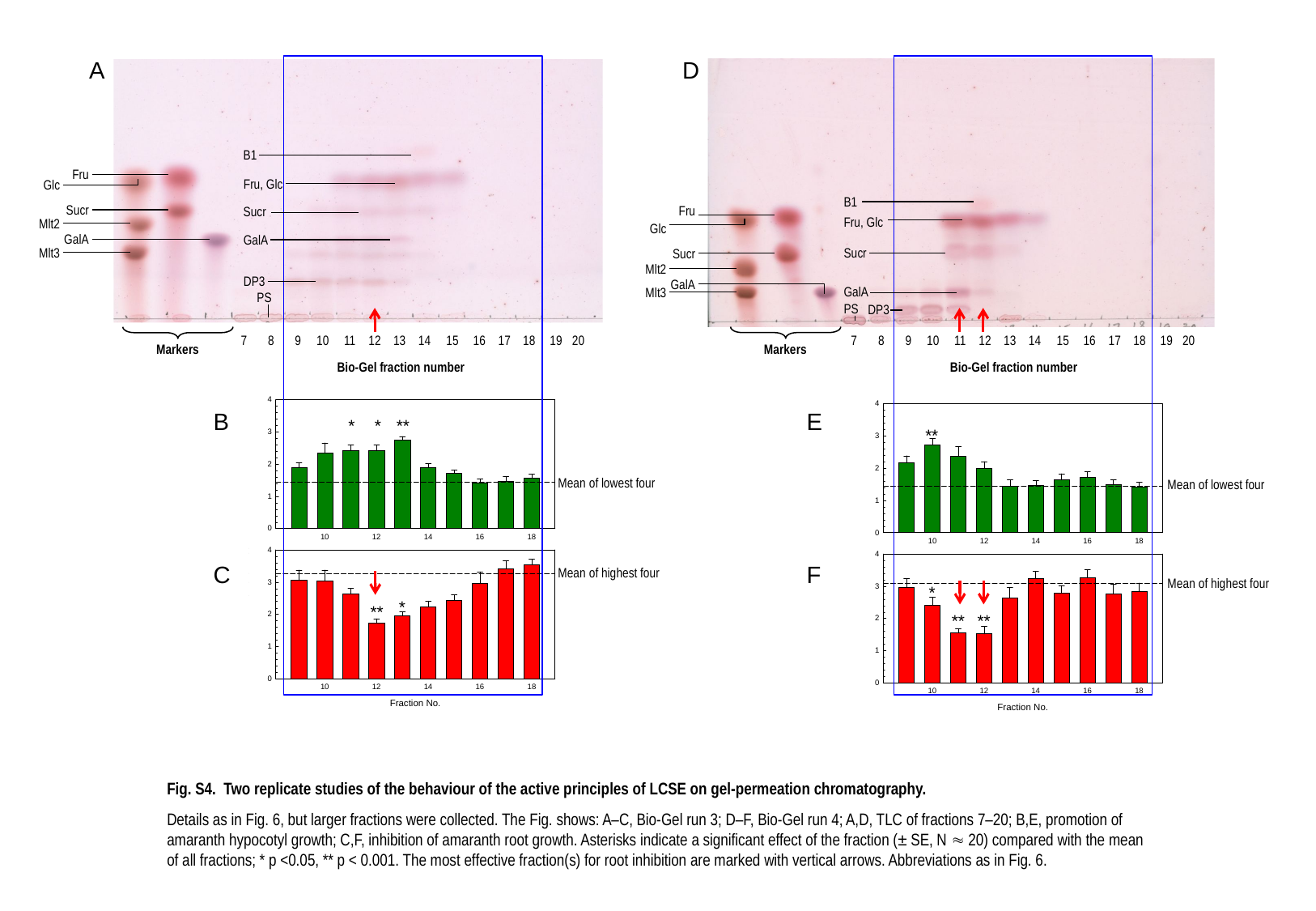

A
D
B1
Fru
Fru, Glc
Glc
B1
Sucr
Fru
Sucr
Fru, Glc
Mlt2
Glc
GalA
GalA
Mlt3
Sucr
Sucr
Mlt2
DP3
GalA
GalA
Mlt3
PS
PS
DP3
7
8
9
10
11
12
13
14
15
16
17
18
19
20
7
8
9
10
11
12
13
14
15
16
17
18
19
20
Markers
Markers
Bio-Gel fraction number
Bio-Gel fraction number
B
E
*
*
**
**
Mean of lowest four
Mean of lowest four
C
F
Mean of highest four
Mean of highest four
*
*
**
**
**
Fig. S4. Two replicate studies of the behaviour of the active principles of LCSE on gel-permeation chromatography.
Details as in Fig. 6, but larger fractions were collected. The Fig. shows: A–C, Bio-Gel run 3; D–F, Bio-Gel run 4; A,D, TLC of fractions 7–20; B,E, promotion of amaranth hypocotyl growth; C,F, inhibition of amaranth root growth. Asterisks indicate a significant effect of the fraction (± SE, N  20) compared with the mean of all fractions; * p <0.05, ** p < 0.001. The most effective fraction(s) for root inhibition are marked with vertical arrows. Abbreviations as in Fig. 6.
